# Cognitive justice and interculturality: the pioneering nature of the professional master’s program for indigenous nurses in Amazonas

**DOI:** 10.1590/1980-220X-REEUSP-2026-0281en

**Published:** 2026-08-31

**Authors:** Rizioléia Marina Pinheiro Pina, Esron Carvalho Soares Rocha, Hadelândia Milon de Oliveira, Silvia Regina Secoli

**Affiliations:** 1 Universidade Federal do Amazonas, Escola de Enfermagem de Manaus, Manaus, AM, Brazil.; 2 Universidade de São Paulo, Escola de Enfermagem, São Paulo, SP, Brazil.

The creation of a master’s program specifically designed for Indigenous nurses from the Upper Rio Negro region in the state of Amazonas—through the Graduate Program in Nursing in the Amazonian Context (PPGENF-MP) at the Manaus School of Nursing of the Federal University of Amazonas (UFAM)—represents a historic and political milestone, at the intersection of advanced education, equity, and recognition.

Located in one of the world’s most socio-culturally diverse regions, the Amazon is home to peoples who have their own systems of care and complex cosmologies, and historically have faced persistent inequities regarding access to education and autonomy in care management. Thus, conceiving, designing, and organizing nurse education at the graduate level implies recognizing the centrality of territory, language, and cultural practices as constitutive dimensions of the educational process and contributing to the reduction of health inequities among indigenous populations.

Within the scope of the Coordination for the Improvement of Higher Education Personnel (CAPES), the professional nature of this program confers upon it strategic potential for local socio-health transformation, operationalized through Technical and Technological Products (PTT in Portuguese), which transform scientific knowledge into practical solutions to meet society’s demands.

In remote and culturally diverse contexts, the PTTs—which are geared toward solving concrete problems in the workplace—transcend mere technical application and serve as mediating tools for the development of hybrid care models. These products are not only capable of adapting evidence-based protocols but, above all, of validating traditional diagnosis and the temporal dimensions of local cosmologies as inseparable aspects of care management. Thus, PTTs can integrate biomedical rationality with social technologies and intercultural protocols, emerging as instruments of cognitive justice that institutionalize oral history, community experience, and territorial praxis as legitimate sources of scientific knowledge.

More than simply training master’s-level nursing professionals, the program aims to strengthen Indigenous leadership so that these professionals can coordinate health care based on their own cosmologies, while simultaneously navigating the formal health care system critically. This approach goes beyond “improvisation”—often a response to a lack of resources and specific training^(1)^—by promoting a transition toward strategic and scientific planning led by Indigenous peoples themselves.

In this process, the institutional role requires a commitment to cognitive justice and decolonization. Historically, health education has been complicit in maintaining colonial structures and systems of privilege that exclude traditional knowledge^(2)^.

As a consequence, the course’s innovation lies in the concrete possibility of articulating distinct systems of knowledge, promoting inclusive transformations in the field of health education. The content of the core courses in the professional master’s program has been revised and adapted in light of both Western and Indigenous frameworks, taking into account local specificities and needs.

Interculturality forms the methodological foundation of the courses, in which the concept of “body-territory” is inseparable from the training of Indigenous master’s students. In this context, the challenge is to promote epistemological changes aimed at building “good living” through alliances that recognize Indigenous science and the knowledge of traditional populations as expressions of “intelligent life” in the Amazon^(3)^. This approach also enables the incorporation of broader models of well-being, such as *Sumak Kawsay* (Buen Vivir), originating in Ecuador and Bolivia, which challenges hegemonic and narrow definitions of health by valuing identity, diversity, and ancestral knowledge^(4)^.

From the perspective of the health care system, this master’s program’s proposal aligns directly with the principles of differentiated care and comprehensive care. By addressing comprehensive care, the proposal reaffirms the need for culturally sensitive care, as advocated by Leininger, ensuring that advanced training results in culturally safe practices, greater community engagement, and better outcomes, thereby expanding the response capacity of health teams in these regions^(5,6)^. Additionally, it promotes the production of situated knowledge, which is essential for addressing challenges specific to the Amazon region, such as infectious diseases, the impacts of climate change, and ongoing socio-territorial transformations.

The relevance of this initiative by UFAM’s Graduate Program in Nursing in the Amazonian Context—linked to the strategic project on technology and innovation for intercultural management and care, aimed at integrating indigenous and Western knowledge, and approved under the Agreement between the Federal Nursing Council and CAPES—transcends the Brazilian context. In countries such as New Zealand, Australia, and Canada, there is a growing recognition that reducing health inequities among Indigenous populations depends directly on transforming educational processes. In this regard, international consensus in the field of health education emphasizes the need to incorporate competencies in cultural safety, social determinants, and coloniality into curricula, as well as to increase the presence of Indigenous professionals in health systems^(7)^.

The implementation of this Master’s Program by PPGENF UFAM in the Upper Rio Negro region strategically aligns with the guidelines of the “Mais Saúde Amazônia 2025–2027” Strategic Agenda, enabling the strengthening of the Indigenous Health Care Subsystem (Sasi-SUS) by institutionalizing respect for traditional practices^(3)^.

The impact of this initiative, however, goes beyond the fulfillment of government goals. The program represents a qualitative leap from mere cultural sensitivity to effective cognitive justice and critical interculturality. By validating ancestral knowledge as science, the Upper Rio Negro program positions itself as a paradigmatic milestone in contemporary nursing, capable of spearheading a global agenda that advocates for equity and the epistemological recognition of Indigenous peoples as pillars of advanced care.
