# ERRATUM: Cognitive justice and interculturality: the pioneering nature of the professional master’s program for indigenous nurses in Amazonas

**DOI:** 10.1590/1980-220X-REEUSP-2026-0281eren

**Published:** 2026-10-26

In the article “Cognitive justice and interculturality: the pioneering nature of the professional master’s program for indigenous nurses in Amazonas”, with DOI code number: https://doi.org/10.1590/1980-220X-REEUSP-2026-0281eren, published in the Revista da Escola de Enfermagem da USP, 60:e20260281, 2026, in the page 1:

**Where it was written:**

“Esron Carvalho Soares Rocha”

**Should read:**

“Esron Soares Carvalho Rocha”

Prof. Dr. Maria Amélia de Campos Oliveira

Editor-in-chief

