# Supplementary material for: Cognitive justice and interculturality: the pioneering nature of the professional master’s program for indigenous nurses in Amazonas
Source: Rev Esc Enferm USP. 2026 Aug 31;60:e20260281. doi: 10.1590/1980-220X-REEUSP-2026-0281en (PMC13544431; doi:10.1590/1980-220X-REEUSP-2026-0281en)
Supplement: Translation

# Translation: Justiça cognitiva e interculturalidade: o pioneirismo do mestrado profissional para enfermeiros indígenas no Amazonas

**DOI:** 10.1590/1980-220X-REEUSP-2026-0281pt

**Authors:** Rizioléia Marina Pinheiro Pina, Esron Carvalho Soares Rocha, Hadelândia Milon de Oliveira, Silvia Regina Secoli

**Affiliations:** 1 Universidade Federal do Amazonas, Escola de Enfermagem de Manaus, Manaus, AM, Brasil.; 2 Universidade de São Paulo, Escola de Enfermagem, São Paulo, SP, Brasil.

A criação do curso de mestrado voltado especificamente para enfermeiros indígenas do Alto Rio Negro, Estado do Amazonas, por meio do Programa de Pós-Graduação Enfermagem no Contexto Amazônico, modalidade Mestrado Profissional (PPGENF-MP) da Escola de Enfermagem de Manaus da Universidade Federal do Amazonas (UFAM), representa um marco histórico e político, na interface entre formação avançada, equidade e reconhecimento.

Situada em uma das regiões de maior diversidade sociocultural do mundo, a Amazônia abriga povos com sistemas próprios de cuidado e de cosmologias complexas que, historicamente, enfrentam iniquidades persistentes quanto ao acesso à educação e à autonomia da gestão do cuidado. Desse modo, pensar, criar e organizar a formação de enfermeiros, em nível de pós-graduação, implica reconhecer a centralidade do território, da língua e das práticas culturais como dimensões constitutivas do processo educativo e contribuir para a redução das iniquidades em saúde indígena.

No âmbito da Coordenação de Aperfeiçoamento de Pessoal de Nível Superior (CAPES), a natureza profissional deste programa confere-lhe um potencial estratégico para a transformação sociossanitária local, operacionalizada por meio dos Produtos Técnicos e Tecnológicos (PTT), que transforma conhecimento científico em soluções práticas para atender demandas da sociedade.

Em contextos remotos e culturalmente diversos os PTT, orientados à resolução de problemas concretos do mundo do trabalho, transcendem à mera aplicação técnica, configuram-se como dispositivos de mediação para a construção de cuidados híbridos. Esses produtos não são capazes apenas de adaptar protocolos baseados em evidências, mas, sobretudo, validar o diagnóstico tradicional e a temporalidade das cosmologias locais como dimensões indissociáveis do manejo do cuidado. Assim, os PTT podem integrar a racionalidade biomédica às tecnologias sociais e aos protocolos interculturais, emergindo como instrumentos de justiça cognitiva que institucionalizam a história oral, a experiência comunitária e a práxis territorial como fontes legítimas de saber científico.

Mais do que formar mestres em enfermagem, o curso pretende fortalecer lideranças indígenas, a fim de que os profissionais sejam capazes de articular o cuidado em saúde a partir de suas próprias cosmologias, ao mesmo tempo em que transitam criticamente pelo sistema formal de saúde. Essa abordagem supera o “elogio ao improviso”, frequentemente uma resposta à falta de recursos e de formação específica^(1)^, promovendo uma transição para o planejamento estratégico e científico, protagonizado pelos próprios povos originários.

Neste processo, o papel institucional exige um compromisso com a justiça cognitiva e a descolonização. Historicamente, a educação em saúde tem sido cúmplice na manutenção de estruturas coloniais e sistemas de privilégio que excluem saberes tradicionais^(2)^.

Deste modo, a inovação do curso reside na possibilidade concreta de articulação entre distintos sistemas de saber, promovendo transformações inclusivas no campo da formação em saúde. Os conteúdos das disciplinas matrizes do mestrado profissional foram revisados e ajustados à luz de referenciais tanto ocidentais quanto indígenas, considerando as especificidades e necessidades locais.

A interculturalidade representa a base metodológica das disciplinas, cujo conceito de corpo-território seja indissociável na formação do mestre indígena. Nesse contexto, o desafio é promover mudanças epistemológicas orientadas à construção do “bem viver”, por meio de alianças que reconheçam a ciência indígena e os saberes das populações tradicionais como expressões de “vida inteligente” na Amazônia^(3)^. Essa abordagem possibilita, ainda, a incorporação de modelos ampliados de bem-estar, como o *Sumak Kawsay* (Buen Vivir), originário do Equador e da Bolívia, que desafia definições hegemônicas e estreitas de saúde ao valorizar a identidade, a diversidade e os conhecimentos ancestrais^(4)^.

Na perspectiva do sistema de saúde, a proposta deste MP dialoga diretamente com os princípios da atenção diferenciada e da integralidade do cuidado. Ao dialogar com a integralidade do cuidado, a proposta resgata a necessidade de um cuidado culturalmente sensível, conforme preconizado por Leininger, garantindo que a qualificação avançada resulte em práticas culturalmente seguras e maior adesão das comunidades e melhores desfechos, ampliando-se a capacidade de resposta das equipes de saúde nos territórios^(5,6)^. Adicionalmente, promove-se a produção de conhecimento situado, essencial para enfrentar desafios específicos da região amazônica, como as doenças infecciosas, os impactos das mudanças climáticas e as transformações socioterritoriais em curso.

A relevância desta iniciativa do Programa de Pós-Graduação Enfermagem no Contexto Amazônico da UFAM, vinculado ao projeto estratégico de tecnologia e inovação para a gestão e o cuidado intercultural, voltado à integração entre conhecimentos indígenas e ocidentais, aprovado no âmbito do Acordo entre o Conselho Federal de Enfermagem (Cofen) e CAPES transcende o contexto brasileiro. Em países como Nova Zelândia, Austrália e Canadá, há um reconhecimento crescente de que a redução das iniquidades em saúde indígena depende diretamente da transformação dos processos formativos. Nesse sentido, consensos internacionais na área da educação em saúde enfatizam a necessidade de incorporar competências em segurança cultural, determinantes sociais e colonialidade nos currículos, bem como ampliar a presença de profissionais indígenas nos sistemas de saúde^(7)^.

A implementação deste MP pelo PPGENF UFAM, no Alto Rio Negro converge, de forma estratégica, com as diretrizes da Agenda Estratégica Mais Saúde Amazônia 2025–2027, possibilitando o fortalecimento do Subsistema de Atenção à Saúde Indígena (Sasi-SUS) ao institucionalizar o respeito às práticas tradicionais^(3)^. O impacto desta iniciativa, contudo, transcende o cumprimento de metas governamentais. O curso representa um salto qualitativo da mera sensibilidade cultural para a efetiva justiça cognitiva e interculturalidade crítica. Ao validar saberes ancestrais como ciência, o curso do Alto Rio Negro posiciona-se como um marco paradigmático na enfermagem contemporânea, capaz de capitanear uma agenda global que reivindica a equidade e o reconhecimento epistemológico dos povos originários como pilares do cuidado avançado.

# Translation: Justicia cognitiva e interculturalidad: el carácter pionero de la maestría profesional para enfermeros indígenas en Amazonas

**DOI:** 10.1590/1980-220X-REEUSP-2026-0281es

**Authors:** Rizioléia Marina Pinheiro Pina, Esron Carvalho Soares Rocha, Hadelândia Milon de Oliveira, Silvia Regina Secoli

**Affiliations:** 1 Universidade Federal do Amazonas, Escola de Enfermagem de Manaus, Manaus, AM, Brasil.; 2 Universidade de São Paulo, Escola de Enfermagem, São Paulo, SP, Brasil.

La creación del curso de maestría dirigido específicamente a enfermeros indígenas del Alto Río Negro, en el estado de Amazonas, a través del Programa de Posgrado en Enfermería en el Contexto Amazónico, modalidad de Maestría Profesional (PPGENF-MP) de la Escuela de Enfermería de Manaos de la Universidad Federal de Amazonas (UFAM), representa un hito histórico y político, en la intersección entre la formación avanzada, la equidad y el reconocimiento.

Ubicada en una de las regiones con mayor diversidad sociocultural del mundo, la Amazonía alberga a pueblos con sistemas propios de atención y cosmologías complejas que, históricamente, han enfrentado desigualdades persistentes en cuanto al acceso a la educación y a la autonomía en la gestión de la atención. De este modo, pensar, crear y organizar la formación de enfermeros a nivel de posgrado implica reconocer la centralidad del territorio, la lengua y las prácticas culturales como dimensiones constitutivas del proceso educativo y contribuir a la reducción de las desigualdades en salud indígena.

En el marco de la Coordinación para el Perfeccionamiento del Personal de Nivel Superior (CAPES), la naturaleza profesional de este programa le confiere un potencial estratégico para la transformación sociosanitaria local, que se materializa a través de los Productos Técnicos y Tecnológicos (PTT), los cuales transforman el conocimiento científico en soluciones prácticas para atender las demandas de la sociedad.

En contextos remotos y culturalmente diversos, los PTT, orientados a la resolución de problemas concretos del mundo laboral, trascienden la mera aplicación técnica y se configuran como dispositivos de mediación para la construcción de cuidados híbridos. Estos productos no solo son capaces de adaptar protocolos basados en la evidencia, sino, sobre todo, de validar el diagnóstico tradicional y la temporalidad de las cosmologías locales como dimensiones indisociables de la gestión de la atención. De este modo, los PTT pueden integrar la racionalidad biomédica con las tecnologías sociales y los protocolos interculturales, emergiendo como instrumentos de justicia cognitiva que institucionalizan la historia oral, la experiencia comunitaria y la praxis territorial como fuentes legítimas de conocimiento científico.

Más que formar maestros en enfermería, el curso busca fortalecer los liderazgos indígenas, para que los profesionales sean capaces de articular la atención en salud a partir de sus propias cosmologías, al tiempo que se desenvuelven críticamente en el sistema formal de salud. Este enfoque va más allá del “elogio a la improvisación”, que suele ser una respuesta a la falta de recursos y de formación específica^(1)^, y promueve una transición hacia la planificación estratégica y científica, protagonizada por los propios pueblos originarios.

En este proceso, el papel institucional exige un compromiso con la justicia cognitiva y la descolonización. Históricamente, la educación en salud ha sido cómplice en el mantenimiento de estructuras coloniales y sistemas de privilegio que excluyen los saberes tradicionales^(2)^.

De este modo, la innovación del curso radica en la posibilidad concreta de articular distintos sistemas de conocimiento, promoviendo transformaciones inclusivas en el ámbito de la formación en salud. Los contenidos de las disciplinas principales de la maestría profesional se revisaron y ajustaron a la luz de referentes tanto occidentales como indígenas, tomando en cuenta las especificidades y necesidades locales.

La interculturalidad representa la base metodológica de las disciplinas, cuyo concepto de cuerpo-territorio es indisociable de la formación del maestro indígena. En este contexto, el desafío consiste en promover cambios epistemológicos orientados a la construcción del “buen vivir”, mediante alianzas que reconozcan la ciencia indígena y los saberes de las poblaciones tradicionales como expresiones de la “vida inteligente” en la Amazonía^(3)^. Este enfoque permite, además, la incorporación de modelos ampliados de bienestar, como el *Sumak Kawsay* (Buen Vivir), originario de Ecuador y Bolivia, que desafía las definiciones hegemónicas y estrechas de salud al valorar la identidad, la diversidad y los conocimientos ancestrales^(4)^.

Desde la perspectiva del sistema de salud, la propuesta de esta Maestría Profesional dialoga directamente con los principios de la atención diferenciada y la integralidad de la atención. Al dialogar con la integralidad de la atención, la propuesta resalta la necesidad de una atención culturalmente sensible, tal como lo recomienda Leininger, garantizando que la capacitación avanzada se traduzca en prácticas culturalmente seguras, una mayor aceptación por parte de las comunidades y mejores resultados, ampliando así la capacidad de respuesta de los equipos de salud en los territorios^(5,6)^. Además, se promueve la generación de conocimiento situado, esencial para enfrentar los desafíos específicos de la región amazónica, como las enfermedades infecciosas, los impactos del cambio climático y las transformaciones socioterritoriales en curso.

La relevancia de esta iniciativa del Programa de Posgrado en Enfermería en el Contexto Amazónico de la UFAM, vinculada al proyecto estratégico de tecnología e innovación para la gestión y la atención intercultural, orientado a la integración entre los conocimientos indígenas y occidentales, aprobado en el marco del Acuerdo entre el Consejo Federal de Enfermería (Cofen) y CAPES, trasciende el contexto brasileño. En países como Nueva Zelanda, Australia y Canadá, existe un reconocimiento cada vez mayor de que la reducción de las desigualdades en la salud indígena depende directamente de la transformación de los procesos formativos. En este sentido, los consensos internacionales en el ámbito de la educación en salud enfatizan la necesidad de incorporar competencias en seguridad cultural, determinantes sociales y colonialidad en los planes de estudio, así como de ampliar la presencia de profesionales indígenas en los sistemas de salud^(7)^.

La implementación de este programa de maestría por parte del PPGENF de la UFAM, en el Alto Río Negro, converge de manera estratégica con las directrices de la Agenda Estratégica “Más Salud en la Amazonía 2025–2027”, lo que permite fortalecer el Subsistema de Atención a la Salud Indígena (Sasi-SUS) al institucionalizar el respeto por las prácticas tradicionales^(3)^.

El impacto de esta iniciativa, sin embargo, trasciende el cumplimiento de las metas gubernamentales. El curso representa un salto cualitativo desde la mera sensibilidad cultural hacia la justicia cognitiva efectiva y la interculturalidad crítica. Al validar los saberes ancestrales como ciencia, el curso del Alto Río Negro se posiciona como un hito paradigmático en la enfermería contemporánea, capaz de liderar una agenda global que reivindica la equidad y el reconocimiento epistemológico de los pueblos originarios como pilares de la atención avanzada.
