# Supplementary material for: ERRATUM: Cognitive justice and interculturality: the pioneering nature of the professional master’s program for indigenous nurses in Amazonas
Source: Rev Esc Enferm USP. 2026 Oct 26;60:e20260281er. doi: 10.1590/1980-220X-REEUSP-2026-0281eren (PMC13739455; doi:10.1590/1980-220X-REEUSP-2026-0281eren)
Supplement: Translation

# Translation: ERRATA: Justiça cognitiva e interculturalidade: o pioneirismo do mestrado profissional para enfermeiros indígenas no Amazonas

**DOI:** 10.1590/1980-220X-REEUSP-2026-0281erpt

No artigo “Justiça cognitiva e interculturalidade: o pioneirismo do mestrado profissional para enfermeiros indígenas no Amazonas”, com número de DOI: https://doi.org/10.1590/1980-220X-REEUSP-2026-0281erpt, publicado na Revista da Escola de Enfermagem da USP, 60:e20260281, 2026, na página 1:

**Onde se lia:**

“Esron Carvalho Soares Rocha”

**Leia-se:**

“Esron Soares Carvalho Rocha”

Profa. Dra. Maria Amélia de Campos Oliveira

Editora-chefe

# Translation: ERRATA: Justicia cognitiva e interculturalidad: el carácter pionero de la maestría profesional para enfermeros indígenas en Amazonase

**DOI:** 10.1590/1980-220X-REEUSP-2026-0281eres

En el artículo “Justicia cognitiva e interculturalidad: el carácter pionero de la maestría profesional para enfermeros indígenas en Amazonas”, con número de DOI: https://doi.org/10.1590/1980-220X-REEUSP-2026-0281eres, publicado en la Revista da Escola de Enfermagem da USP, 60:e20260281, 2026, en la página 1:

**Donde se leía:**

“Esron Carvalho Soares Rocha”

**Debe decir:**

“Esron Soares Carvalho Rocha”

Profa. Dra. Maria Amélia de Campos Oliveira

Editora-jefe
